# African swine fever virus RNA polymerase subunits C315R and H359L inhibition host translation by activating the PKR-eIF2a pathway and suppression inflammatory responses

**DOI:** 10.3389/fmicb.2024.1469166

**Published:** 2024-09-24

**Authors:** Saixia Yang, Yiwang Wang, Jifei Yang, Zhancheng Tian, Mengli Wu, Hualin Sun, Xiaoqiang Zhang, Yaru Zhao, Jianxun Luo, Guiquan Guan, Hong Yin, Rongzeng Hao, Qingli Niu

**Affiliations:** ^1^State Key Laboratory for Animal Disease Control and Prevention, College of Veterinary Medicine, Lanzhou University, Lanzhou Veterinary Research Institute, Chinese Academy of Agricultural Sciences, Lanzhou, Gansu, China; ^2^African Swine Fever Regional Laboratory of China (Lanzhou), Gansu Province Research Center for Basic Disciplines of Pathogen Biology, Lanzhou, Gansu, China; ^3^Jiangsu Co-Innovation Center for the Prevention and Control of Important Animal Infectious Disease and Zoonosis, Yangzhou University, Yangzhou, Jiangsu, China

**Keywords:** African swine fever virus, TFIIB, RPB3, C315R, H359L

## Abstract

ASFV C315R is homologous to the transcription factor TFIIB of large unclassified DNA viruses, and H359L is identical to the subunit 3 (RPB3) of eukaryotic RNA polymerase II. The C315R and H359L may play an important role in ASFV replication and transcription. Here, we evaluated the biological function of the *C315R* and *H359L* genes during virus replication *in vitro* and during infection in pigs. Results showed that C315R and H359L are highly conserved among ASFV genotype II strains; quantitative PCR (qPCR) and western blotting analyses revealed that *C315R* and *H359L* are early transcribed genes prior to viral DNA replication, but their protein expression is delayed. The immunofluorescence and western blotting analysis revealed that both proteins localized in the cell cytoplasm and nucleus at 24 h post infection, however, pH359L was mainly detected in the cell cytoplasm. Furthermore, overexpression of pH359L in MA104 cells significantly increased viral titer, RNA transcription levels, and viral protein expression levels, while overexpression of pC315R slightly enhanced ASFV replication. In contrast, siRNA targeting ASFV-H359L or C315R reduced replication efficiency in porcine macrophage culture compared to the parent ASFV-CN/SC/2019, demonstrating that *C315R* and *H359L* genes are necessary for ASFV replication. Finally, the functional role of C315R or H359L on PKR and eIF2α phosphorylation status and SG formation, as well as cytokine production were evaluated. These studies demonstrated that C315R and H359L are involved in virus replication processes in swine and play important roles in ASFV replication.

## Highlights

- African swine fever virus RNA polymerase subunits C315R and H359L plays important roles in ASFV replication.
- C315R and H359L inhibits host translation by activating the PKR-eIF2a pathway.
- C315R and H359L suppress pro-inflammatory response.

## 1 Introduction

African swine fever (ASF) is a highly contagious and lethal hemorrhagic disease affecting domestic pigs, wild boar, and warthogs, with mortality rates that can reach 100%. The disease has spread across five continents—Asia, Africa, North America, Europe, and Australia—causing substantial economic losses worldwide (Zhao et al., 2019). In 2018, a genotype II strain of African swine fever virus (ASFV) was introduced to China, which is a high-virulence strain with fatality rates approaching 100% and has caused devastating socioeconomic losses in China’s pig breeding industry, quickly spreading throughout the country, and severely impacting large-scale pig production (Zhou et al., 2018).

At present, ASF has already caused the deaths of several wild boars in China, making the prevention and control of this disease increasingly severe. Soft ticks of the genus Ornithodoros, which serve as a biological vector of virus, have played a significant role in the epidemiology of the disease (Gaudreault et al., 2020). Although there are no reports regarding ticks transmitting ASFV in China, but previous study has confirmed that their presence can facilitate the formation of natural foci. If the virus enters the wild boar-soft tick forest cycle, which will become one of the main obstacles to eradicating ASF (Pereira De Oliveira et al., 2020). Currently, there are some reports indicate that ASFV has infected wild boars in Jilin Province, Heilongjiang Province, Hunan Province, Shaanxi Province, and Hubei Province. The ASFV has appeared in the fields with naturally mutated strains of genotype II, genotype I, and recombinant strains of genotype I/II, complicating the disease’s occurrence and prevalence in China (Sun et al., 2021a; Zhao et al., 2023; Sun et al., 2021b). The complex situation of the co-existence of these mutated and new strains presents significant challenges for ASF prevention and control in China.

ASF is caused by a nucleocytoplasmic large double-stranded DNA virus (ASFV, NCLDVs, 170–193 kb), the sole member of the *Asfarviridae* family, genus *Asfivirus*, and the only known DNA arbovirus that depends on soft ticks of the genus *Ornithodoros* for transmission (Matsuyama et al., 2020). The ASFV particle is enveloped, with an overall icosahedral morphology and an average diameter of around 200 nm. It features a continuous outer lipid envelope, an icosahedral protein outer capsid, an inner lipid envelope, a thick protein core shell, and an internal genome nucleoid. Each layer contains many proteins, but only few proteins have been studied at the structural level (Andrés et al., 2020). The ASFV genome comprises approximately 150–170 open reading frames (ORFs), which are closely spaced with short upstream promoter sequences and are read from both DNA strands (Wang et al., 2021). The variation in the number of genes and genome length across ASFV strains are mainly due to the acquisition or loss of ORFs from the multigene family (MGF). ASFV particles encode about 68 functionally known structural proteins and enzymes, and more than 100 non-structural proteins involved in regulating viral DNA replication, mRNA transcription, nucleotide metabolism, and DNA repair, as well as modulating host cell functions and facilitating viral immune escape (Chen et al., 2021; Reis et al., 2017; Galindo and Alonso, 2017). However, 34% of viral proteins remain functionally uncharacterized, which limits the identification of replication and virulence related genes of ASFV and the development of effective antiviral treatments (Alejo et al., 2018).

Gene transcription is an essential biological process that leads to RNA production from the DNA template. In eukaryotes, three kinds of RNA polymerases (RNAP I, II and III), responsible for catalyzing RNA synthesis, are key enzymes in the transcription process. RNAP II, composed of 8–14 subunits and transcribes mRNAs and some small nuclear RNAs (O’Brien and Ansari, 2021; Nikolov and Burley, 1997). This polymerase requires gene-specific transcription factors to initiate transcriptional function (O’Brien and Ansari, 2021; Nikolov and Burley, 1997).

The ASFV replication begins in the nucleus of the host cell and later completed in the cytoplasm of the infected host cell. The virus encodes a complex eukaryotic-like 9-subunit RNAP transcription systems, including homologs of seven (RPB1, 2, 3, 5, 6, 7, 10) of the 14 of eukaryotic Pol II subunits, TFIIB and TFIIS, which is characterized by its relative independence from the host cell’s transcriptional machinery for replication (Iyer et al., 2006; Cackett et al., 2020b). Furthermore, previous study has shown that this relative independence transcription mechanism of ASFV from the host cells was interrelated to its encoding at least 20 genes involved in transcription, mRNA modification and other vital lifecycle processes, such as DNA polymerases, DNA helicases, topoisomerases for DNA manipulation, transcription factors involved in transcription initiation, intermediate and elongation (Rodríguez and Salas, 2013).

A major challenge in developing effective antiviral treatments for ASFV is the limited understanding of the function of viral proteins in DNA replication, gene transcription and translation. Therefore, it is crucial to analyze the functional roles of these proteins in ASFV to understand the virus’s replication process *in vitro* and its virulence *in vivo*. This understanding could accelerate the development of new ASF vaccines and antiviral drugs. The ASFV genes *C315R* and *H359L*, remarkably similar to the eukaryotic RNAP II transcription factor (TFIIB) and subunit (RPB3) were identified using functional genomics (Iyer et al., 2006), but their biological characteristics and functions remain unknown.

The aim of our study is to apply a serious of molecular biology technologies to characterize the biological characteristics and phylogenetic relationships of C315R and H359L among different ASFV isolates. We evaluated the dynamics of transcription and expression patterns of these genes *in vitro* or *ex vivo*, as well as their intracellular distribution. We also discussed the implications of C315R and H359L gene evolution, expression kinetic at the protein and RNA level, their localization in infected cells, and their impact on virus replication by knockdown or overexpression. Finally, we assessed the functional role of C315R or H359L in PKR and eIF2α phosphorylation status and stress granule (SG) formation, as well as cytokine production. Altogether, our study highlights the function of ASFV-C315R or-H359L and provides a novel target for the development of anti-ASFV strategies that could block the infectious cycle of ASFV.

## 2 Materials and methods

### 2.1 Biosafety and ethics statement

All experiments involving live ASFV were performed in a biosafety level-3 (BSL-3) facility at the Lanzhou Veterinary Research Institute (LVRI), Chinese Academy of Agriculture and Sciences (CAAS) accredited by China National Accreditation Service for Conformity Assessment (CNAS) and approved by the Ministry of Agriculture and Rural Affairs of China. The animal treatments and sample collection were approved by the Animal Ethics Committee of the LVRI, CAAS (LVRIAEC-2023-043). All animals were handled in accordance with the Animal Ethics Procedures and Guidelines of the People’s Republic of China.

### 2.2 Cells and viruses

Primary alveolar macrophages (PAMs) were harvested from 30-day-old health pigs and cultured in RPMI 1640 medium (Gibco, 11,875,085) containing 10% fetal bovine serum (10,091,148, Gibco) and 1% penicillin–streptomycin (V900929, Sigma) at 37°C in a humidified incubator with 5% CO2. 3D4/21 (Immortalized porcine alveolar macrophage, IPAM) and PK15 cells were cultured in Dulbecco’s Modified Eagle Medium (DMEM) (Gibco, Grand Island, NY) supplemented with 10% FBS (PAN-Biotech, Aidenbach, Germany). HEK-293 T cells were cultured in DMEM medium (DMEM; Gibco, China) containing 10% fetal bovine serum (FBS; Gibco, 16,000–044), 1% penicillin, and streptomycin (Gibco, 1,514,012) and African green monkey kidney epithelial cells (MA104) were cultured in MEM medium (Gibco, A1049001) containing 10% fetal bovine serum and 1% penicillin and streptomycin. ASFV CN/SC/2019 was isolated, identified, and maintained in LVRI-BSL-3 of the African Swine Fever Regional Laboratory of China (Lanzhou).

### 2.3 Antibodies and reagents

The antibodies used in this study are detailed as follows: Horseradish peroxidase (HRP)-conjugated goat anti-rabbit or-mouse secondary antibody (SA00001-2; SA00013-3), anti-G3BP1 polyclonal antibody (13057-2-AP) and ani-PKR Rabbit Polyclonal Antibody (18244-1-AP) were purchased from Proteintech. Anti-eIF2α Rabbit mAb (A21221), anti-Phospho-eIF2α-S51 Rabbit mAb (AP0692) and anti-Phospho-PKR Rabbit mAb (AP1134) were purchased from Abclonal. Anti-*β*-tubulin (32–2,600), goat anti-rabbit IgG (H + L) (Alexa Fluor 488, A32731) were purchased from Invitrogen. Anti-p30, anti-p72, anti-C315 and anti-H359l rabbit polyclonal antibodies were prepared and preserved by the African Swine Fever Regional Laboratory, China (Lanzhou), Lanzhou Veterinary Research Institute (LVRI) of the Chinese Academy of Agricultural Sciences. Cytosine arabinoside (AraC S18123) was purchased from Shanghai Yuanye Bio-Technology Co., Ltd.

### 2.4 Plasmid construction and transfection

The ASFV *C315R* or *H359L* gene was amplified from ASFV infected PAMs and cloned into C-terminally tagged pcDNA-3.1 vector. For 3D4/21(IPAM) cells, the special targeting Macrophage cells reagent, which is jetPEI-Macrophage Polyplus Transfection (PT-103-05 N) was used for transfection experiments. For other cell lines, jetPRIME Polyplus Transfection (101000046) were used, according to the manufacturer’s instructions. The cells, at approximately 80% confluence, were transfected and then harvested for analysis 24 h later using various methodologies.

### 2.5 RNA interference assay

Three double-stranded siRNAs targeting *C315R* or *H359L* transcripts were designed and synthesized by Shanghai Gemma Gene to knockdown endogenous *C315R* or *H359L* expression. The siRNA sequences used in the study are shown in Supplementary Table S2. For knockdown of C315R or H359L, siRNA control or siRNA-*C315R* or-H359L was transfected into PAMs using Lipofectamine 2000 (R0532, Invitrogen). The knockdown efficiency of the target genes was validated 24 h post-transfection by qRT-PCR and Western blot.

### 2.6 HAD_50_ assay

Primary PAM cells were seeded in 96-well plates at eight replicates and inoculated with 10-fold serial dilutions of ASFV (CN/SC/2019). Prior to observing ASFV-induced cytopathic effects in PAMs, 20 μL of freshly prepared 1% pig erythrocytes in saline solution (diluted to 1/100 in 1 XPBS) was added to each well. The cells were incubated at 37°C, and ASFV was quantified by observing the formation of characteristic rosettes through hemadsorption (HAD) of erythrocytes around infected cells. HAD activity was monitored for 5–7 consecutive days post-inoculation, and the 50% HAD dose (HAD_50_) was calculated using Reed and Muench (1938) method.

### 2.7 Cell viability assay

Cell viability was assessed using CCK-8 kit (Cat K1018, APExBIO, United States). Briefly, cells (2 × 10^4^ cells per well) were seeded in 96-well plates and cultured for 24 h prior to treatment with varying concentrations of plasmids (0.25–2 μg) or a single concentration. Each experiment included three replicates, and an empty vector control was included. The treated cells were incubated for 24 h at 37°C in 5% CO_2_. Subsequently, 10% CCK-8 solution (Bimake, B34304) was added to each well and incubated for 1–4 h at 37°C. Absorbance was measured at 450 nm using a microplate reader, and cell viability was calculated using the following formula: cell viability (%) = [(OD plasmids – OD blank)/(OD control−OD blank)] × 100.

### 2.8 RT-qPCR

The 3D4/21 cells were seeded in 12-well plates and transfected with indicated plasmids (2 μg) for 12 hpt, following ASFV infection for 12hpi. Total RNA was extracted from different cell samples using Trizol reagent (Invitrogen, 15,596,018) according to the manufacturer’s instructions. cDNA was synthesized from RNA using the PrimeScript^TM^ RT Reagent Kit with gDNA Eraser (Takara Bio Inc., RR047A) and used as a template for amplification analysis of all target genes. RT-PCR was performed with the One Step Prime Script RT PCR Kit (Takara, RR064A). The PCR primer sequences used in the reaction are listed in Supplementary Table S2. Relative expression of each designated gene was calculated by the 2^−△△Ct^ method and normalized with GAPDH.

### 2.9 Western blotting analysis

Cells, with or without ASFV infected, were harvested and lysed in 150 μL RIPA buffer (Thermo Fisher Scientific, 89,900) supplemented with protease inhibitor cocktail (Thermo Fisher Scientific, 87,786) for 3 h on ice. Then the whole protein extracts were then pelleted and separated by SDS-PAGE and transferred to a polyvinylidene difluoride membrane (Cytiva, 10,600,023), blocked with 5% (w/v) skimmed milk. Next, the membrane was incubated with primary antibodies at 4°C for overnight. Then it was incubated with corresponding secondary antibodies conjugated to HRP at room temperature for 1 h. Finally, the protein levels were detected using ECL reagents (Thermo Fisher Scientific, 32,106) and quantified using Quantity One software (Bio-Rad Laboratories).

### 2.10 Immunofluorescence and confocal microscopy

The PAMs or 3D4/21 cells were seeded in laser confocal dishes and treated in different conditions. Then, the cells were washed twice with pre-cooled PBS and then fixed with 4% paraformaldehyde (BBI, E672002-0500) for 30 min at room temperature, washed three times with pre-cooled PBS, and incubated with 4% bovine serum albumin (BSA, Sigma Aldrich, A9647) at room temperature for 1 h, followed by three times with pre-cooled PBS, then permeabilized with 1% Triton-X 100 (Sigma Aldrich, T8787) for 20 min at room temperature, followed by blocking with 5% BSA in PBS for 1 h. After that, cells were incubated with a certain fold dilution of the corresponding primary antibodies at 4°C overnight, then incubated with fluorochrome-conjugated secondary antibodies for 1 h in the dark. Meanwhile, cells were incubated with DAPI (Invitrogen, P36941) for nuclear staining for 10 min at room temperature. Last, the fluorescence signals were detected with a TCS SP8 confocal fluorescence microscope (Leica, Germany).

### 2.11 Statistical analysis

Statistical analysis of all data was performed using Prism 8.0 (GraphPad Software, Inc.). Two-tailed *p*-values were denoted, and *p*-values <0.05 was statistically significant (*p < 0.05, **p < 0.01, ***p < 0.001, and ****p < 0.0001). Statistical comparison between groups was performed using paired or non-paired *t-*test. Quantitative data displayed in all figures are expressed as means ± SD (Represented as error bars).

## 3 Results

### 3.1 pC315R or pH359L amino acid sequences are conserved among different isolates in same genotype of ASFV

**Figure 1:**
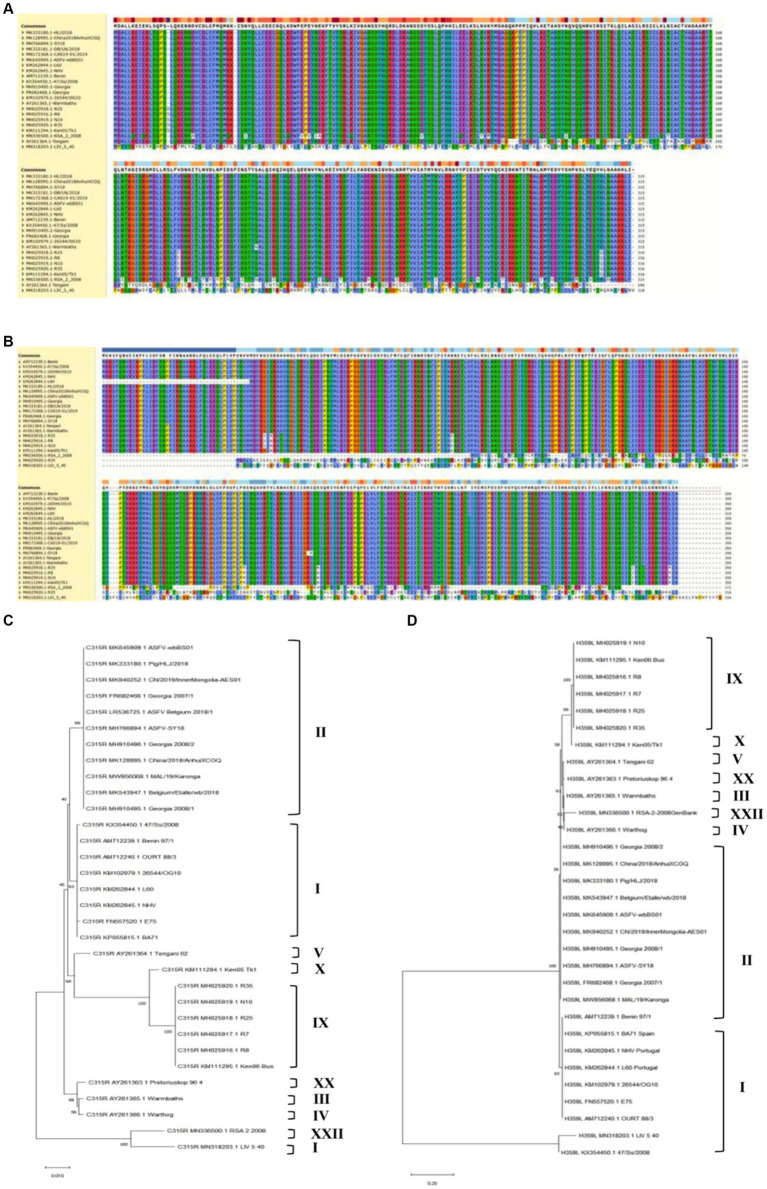
Biological characteristics of the ASFV-C315R and H359L. Multiple amino acid sequence alignment of pC315R **(A)** and pH359L **(B)** of 22 ASFV isolates using the software ClustalW 2.0.12: Clustalw: multiple alignment **(A,B)**. Phylogenetic tree of the amino acid sequences of ASFV pC315R and pH359L and of all known 33 different members of the pC315R or pH359L in ASFV. The tree was inferred using the neighbor joining method of MEGA 10.0 software, bootstrap values are shown at each branch point **(C,D)**.

To analyze the genetic evolution of pC315R or pH359L amino acid sequences in ASFV, we selected 36 isolates from 10 genotypes deposited in GenBank. The percent identity among these isolates was compared (Supplementary Table S1), and the corresponding alignment of pC315R or pH359L amino acid sequences from 22 isolates was conducted by Clustal W method, respectively, (Figures 1A,B). Subsequently, we constructed phylogenetic trees with 32 isolates for pC315R and 31 isolates for pH359L available in GenBank (Figures 1C,D). The result revealed that pC315R and pH359L amino acid sequences are notably conserved across the genotypes, particularly in genotypes I and II, where similarity exceeded 99%. Notably, the maximum identity observed among genotype II isolates was 100%, while the minimum identity to SY18 isolate, which is first reported in China, was 99.9% for pC315R based on amino acid sequences. In contrast, pH359L displayed higher identity across genotype II isolates in both nucleotide and amino acid sequences.

The analysis of homology across different genotypes showed that the minimum identity of these isolates to SY18 was 89% for nucleotides and 92.3% for amino acids in C315R. For H359L, the homology is slightly lower at 83.8% for nucleotides and 86.4% for amino acids, respectively (Supplementary Table S1). As expected from the similarities already described above, the phylogenetic tree, based on the pC315R amino acid sequence, distinguished the 10 genotypes into separate branches, typically clustering isolates from the same genotype within the same clades. Notably, the pC315R of the SY18, HLJ/2018 and Inner Mongolia 2019 strains from China grouped with the Georgia 2007/1, Belgium 2018 and ASFV-wbBs01 strains in the same branch. It is worth noting that LIV_5_40 isolate from genotype I formed a distinct clade with genotype XXII, while isolates from genotype III and IV clustered with genotype XX. In terms of pH359L conservation, it showed higher conservation than the pC315R, with sequences from different genotypes forming a sister clade with each other, and two isolates from genotype I branching off separately. This detailed genetic analysis underscores the high degree of conservation in certain ASFV gene sequences and illustrates the complex phylogenetic relationships among different genotypes, providing crucial insights for future studies on ASFV diversity and evolution.

### 3.2 The abundance of *C315R* and *H359L* genes expression were the highest in spleen of dead pigs acutely infected by ASFV

**Figure 2:**
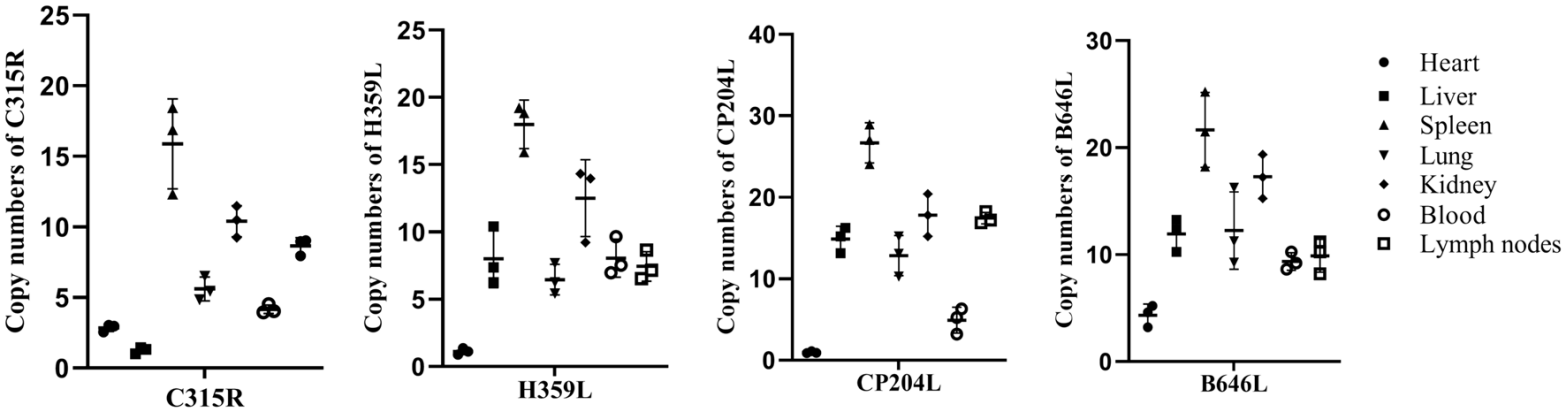
ASFV-*C315R*, -*H359L*, -*CP204L*, and-B646L gene detection in different tissues of ASFV infected pigs by qPCR. DNA was extracted from blood, heart, spleen, liver, lung, lymph nodes and kidney samples from acutely infected pigs. ASFV *C315R*, *H359L*, *CP204L* and *B646L* gene copy numbers were quantified by qPCR assays, and the error bars represent the standard deviation among replicates. Data are shown as the mean ± SD based on three independent experiments.

To determine the abundance of C315R and H359L expression *in vivo*, total genomic DNA was extracted from blood, as well as homogenates from the heart, liver, spleen, lung, kidney, and lymph nodes of dead pigs acutely infected by ASFV. The quantification was performed using qPCR on a CFX96 Touch Real-Time PCR instrument (Bio-Rad, United States) with Quick 96-Well Plates. As shown in Figure 2, the DNA copy numbers of the *C315R* gene were generally consistent with those of the *CP204L* gene. The highest abundance of *C315R* was observed in the spleen, followed by the kidney, lymph nodes, lung, and blood, while its expression was relatively low in the liver and heart, particularly in the liver where it was the lowest expression of *C315R* gene, compared with *H359L*, *CP204L* and *B646L* genes. Conversely, the DNA copies of H359L in the blood and various tissues (heart, liver, spleen, lung, kidney, and lymph nodes) exhibited similar patterns to those of B646L expression with the lowest expression level of H359L occurring in the heart.

### 3.3 *C315R* and *H359L* genes are transcribed early during ASFV replication, encoding for early (C315R) and intermediate protein (H359L)

**Figure 3:**
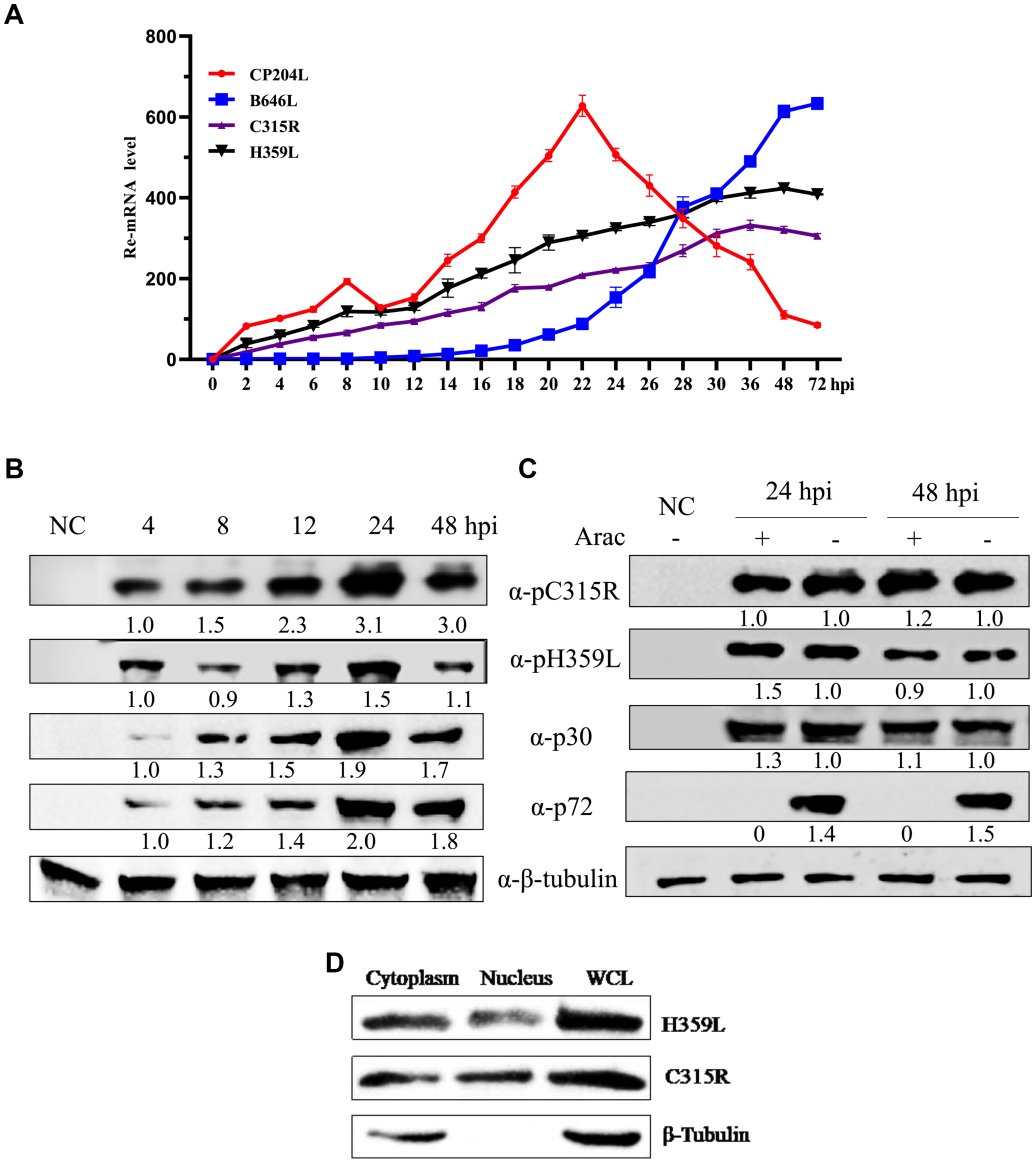
ASFV-*C315R* and *H359L* gene transcriptional and expressional dynamics. Relative mRNA level of the ASFV CN/SC/2019-infected primary porcine alveolar macrophages (PAM, MOI of 1) at 0–72 hpi were detected by quantitative reverse transcription-PCR analyses using primers against *C315R*, *H359L*, *CP204L*, and *B646L*. Results are shown as mean ± standard error of the number of transcripts of each viral gene normalized with reference gene GAPDH mRNA levels. Three independent experiments were performed in duplicate **(A)**. Western blot analysis of 4 proteins expressional kinetics in PAMs harvested at different times after infection with ASFV CN/SC/2019 (MOI of 1), and proteins extracted from uninfected cells as negative control **(B)**. The PAMs infected with ASFV CN/SC/2019 isolate (MOI of 1) were harvested in the presence or absence of an inhibitor of DNA replication (AraC) performed after an initial viral adsorption period (1 h) and cells were collected at 24 or 48 hpi **(C)**. ASFV C315R and H359L proteins localized in both cytoplasm and nucleus in PAMs. PAMs were infected with ASFV CN/SC/2019 isolate for 24 h, nuclear and cytoplasmic extraction were performed with NEPER Nuclear and Cytoplasmic Extraction Reagents (Thermo Fisher Scientific, Waltham, MA, United States), according to the manufacturer’s instructions. The expression and localization of C315R and H359L protein were evaluated by and western blot by using C315R and H359L specific antibodies **(D)**.

To investigate the transcription kinetics of the *C315R* and *H359L* genes, Porcine Alveolar Macrophage (PAM) cells infected with ASFV at a multiplicity of infection (MOI) of 1 were sampled at various time points post-infection. Total RNA and protein were extracted for analysis using real-time quantitative PCR (qPCR) and Western blot. The mRNA levels of C315R and H359L were monitored at multiple time points ranging from 0 to 72 h post-infection (hpi), showing detectable transcription from as early as 2 hpi throughout the entire duration of the study. This pattern was consistent with the early transcribed control gene *CP204L* (Figure 3A).

The transcription of the late control gene *B646L*, however, commenced at 16 hpi, indicating its role in later stages of viral replication (Figure 3A). Western blot analyses tracked the expression of ASFV proteins p30 and p72, which are markers for early and late stages of the viral replication cycle, respectively. The results indicated that p30 was expressed around 4 hpi and p72 around 24 hpi. Interestingly, the expression of pC315R was detected at 4 hpi, aligning with the early expressed protein p30, whereas pH359L was detected at 12 hpi, and expressed between the early expressed p30 and the late expressed p72, suggesting its role as an intermediate protein (Figure 3B).

Further analysis using the DNA replication inhibitor AraC by Western blot analysis demonstrated that the expression levels of pC315R, pH359L, and p30 were unaffected by the presence of AraC, indicating that their expression is prior to viral DNA replication. In contrast, the expression of the control protein p72 was almost completely inhibited in the presence of AraC, confirming its classification as a late protein after viral DNA replication (Figure 3C). These results substantiate the roles of pC315R and pH359L as early and intermediate proteins, respectively, within the ASFV replication cycle. Additionally, the localization of exogenously introduced HA-tagged C315R and H359L proteins was confirmed to be primarily in the cytoplasm (Supplementary Figure S1). Western blotting further validated these findings, indicating a major cytoplasmic presence for both C315R and H359L (Figure 3D). Collectively, these results suggest that C315R and H359L proteins localize in both the cytoplasm and nucleus, with a notable preference for the cytoplasm.

### 3.4 pC315R and pH359L are important proteins for ASFV infection and promote the replication of ASFV in both PAM and MA104 cells

**Figure 4:**
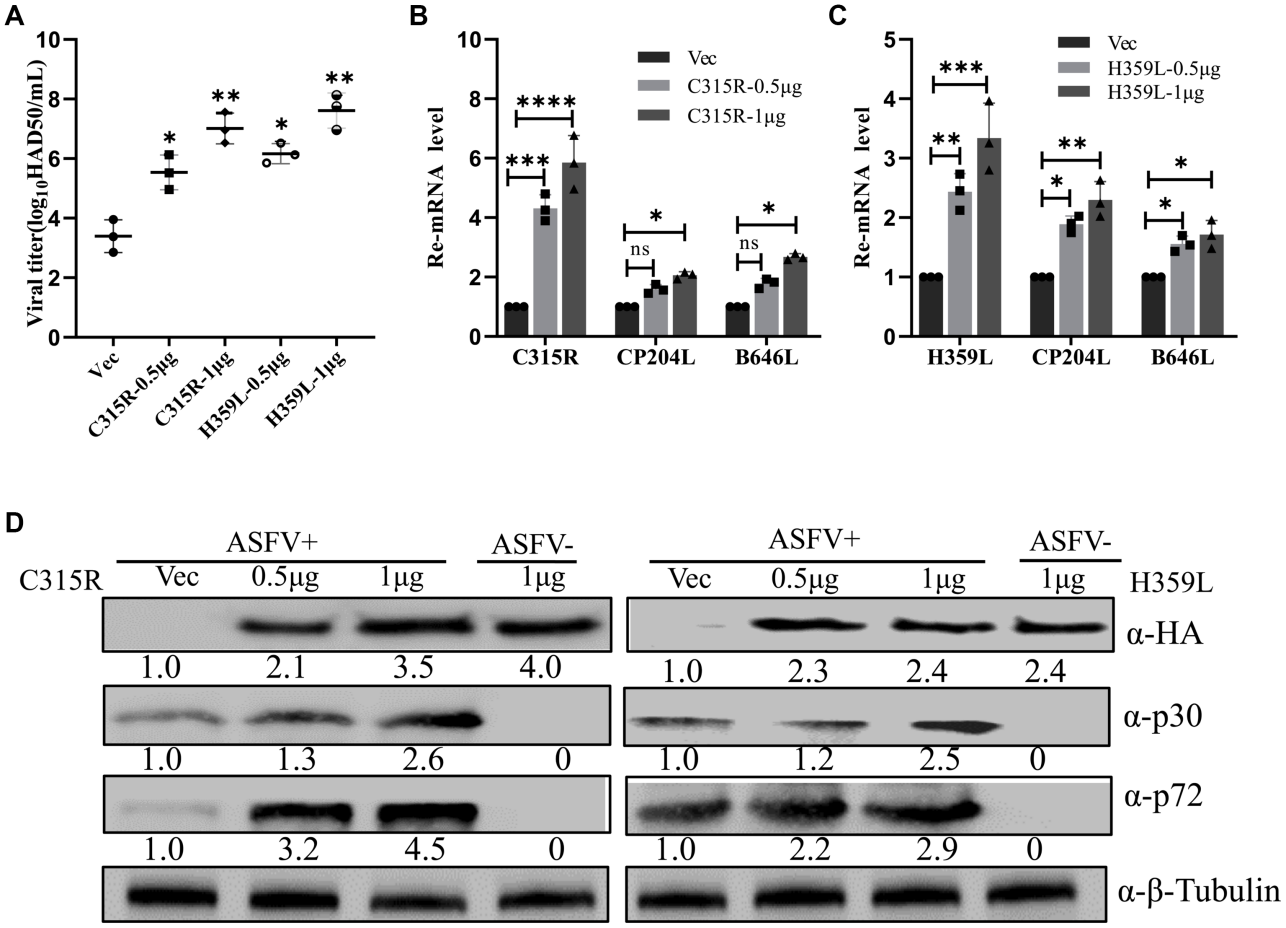
Overexpression of ASFV pC315R and pH359L promotes virus replication and progeny production. MA104 cells were transfected with plasmids encoding ASFV C315R or H359L or with vector pCDNA3.1. At 24 h post-transfection. Cells were infected with ASFV CN/SC/2019 (MOI of 1) for 24 h. A viral titer **(A)**, gene transcriptional level **(B,C)** and viral protein expression **(D)** in pC315R or pH359L-overexpressed cells were detected. The ratio of C315R or H359L or P30 or P72 in C315R or H359L-transfected cells, to vector pCDNA3.1-transfected cell, were shown.

To elucidate the role of ASFV C315R and H359L in viral replication, an overexpression assay was performed. Initially, pFlag-C315R or pFlag-H359L were transfected into MA104 cells at increasing doses for 24 h, followed by ASFV infection (MOI = 1). Subsequent analyses using HAD_50_ experiments, RT-qPCR, and Western blotting demonstrated that overexpressing C315R or H359L significantly enhanced ASFV titer and increased the mRNA and protein levels of *CP204L* (p30) and *B646L* (p72) in a dose-dependent manner, compared to the control group (Figures 4A–D). The effect of C315R or H359L overexpression was further confirmed in various cell lines via Western blotting (Supplementary Figure S2). These results suggest that ASFV C315R and H359L notably promote ASFV virus replication.

**Figure 5:**
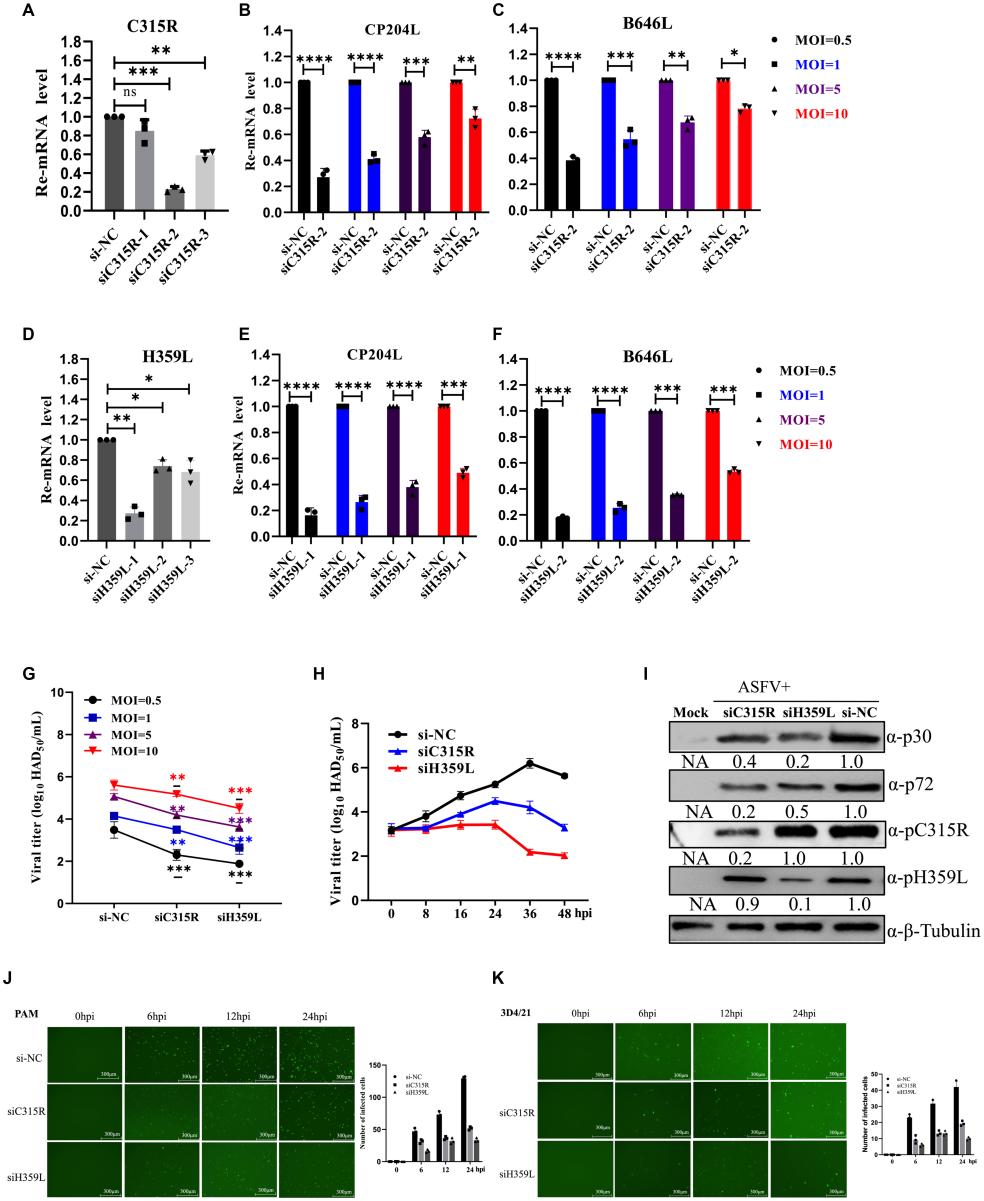
Downregulation of ASFV C315R and H359L disrupts ASFV DNA replication and progeny production. PAMs cells were transfected with three double-stranded siRNAs targeting C315R or H359L (50 nM) for 12 h and then harvested for RNA extraction, the interfered effect was detected by Q-PCR **(A,D)**. PAMs cells were transfected with siRNA targeting C315R or H359L (50 nM) for 4 h, and then infected with ASFV CN/SC/2019 (MOI = 0.5–10) for 24 h. The expression of ASFV early gene *CP204L* and late gene *B646L* were detected by Q-PCR in C315R or H359L-depleted cells (MOI = 0.5–10) **(B–F)**. PAMs cells were transfected with siRNA targeting C315R or H359L or si-NC and infected with ASFV CN/SC 2019 (MOI = 0.5–10) for 12 h or infected with different time points (8, 16, 24, 36, and 48 hpi, respectively). Viral titers were assessed by the HAD_50_ assay **(G,H)**. PAMs cells were transfected with siRNA targeting C315R or H359L or si-NC and infected with ASFV CN/SC/2019 (MOI = 0.5–10) for 24 h, the cell lysis was collected and subjected to immunoblot analysis to evaluate the expression of P30, P72, C315R, or H359L proteins. The ratio of C315R or H359L or P30 or P72 in C315R or H359L-depleted cells, to vector si-NC treated cells, were shown **(I)**. PAMs or D34/21 cells were transfected with siRNA targeting C315R or H359L or si-NC and infected with ASFV-GFP-CN/SC/2019 (MOI = 1) for 0, 6, 12, and 24 hpi, respectively **(J,K)**. Viral replication was analyzed by fluorescent microscopy and GFP positive cells were analyzed by Image J. Scale bar, 300 μm. Data were shown as mean ± SD based on three independent experiments. Statistical significance is denoted by asterisks (*p < 0.05, **p < 0.01, ***p < 0.001, ****p < 0.0001 determined by two-tailed Student’s *t*-test. ns, no significance).

To further explore the role of *C315R* and *H359L* in ASFV replication, three siRNAs targeting *C315R* or *H359L* were designed and synthesized and their knockdown efficiency on PAM cells was assessed. SiC315R-2 and siH359L-1 were chosen for subsequent experiments due to their effective knockdown in PAM cells, as demonstrated by RT-qPCR (Figures 5A,D). Subsequently, PAM cells were transfected with siC315R-2 or siH359L-1 for 24 h, followed by ASFV infection (MOI = 1) for 24 hpi, the viral RNA, viral loads and the viral protein levels were detected. The results indicated that a significant reduction in ASFV-*CP204L* and ASFV-*B646L* gene transcription levels by 70–30% and 60–20% in *C315R*-depleted cells (MOI = 0.5–10), and 80–50% and 78–40% in *H359L*-depleted cells, respectively, compared to the control (Figures 5B,C,E,F). Viral yields also showed a statistically significant reduction across various MOIs and infection time points in PAM cells (Figures 5G,H). Furthermore, ASFV p30 protein expression in C315R and H359L-depleted cells was decreased with about 5 times for siH359L, about 2 times for siC315R, while ASFV p72 protein expression was decreased with about 2 times for siH359L, about 5 times for siC315R, compared to the control group (Figure 5I). The replication of ASFV was significantly decreased in C315R or H359L knockdown cells following ASFV-GFP infection from 6 to 24 hpi in both PAM (Figure 5J) or 3D4/21 cells (Figure 5K), as detected by IFA. This indicates that targeting *C315R* and *H359L* with siRNAs significantly inhibits ASFV DNA replication and progeny production, particularly with the loss of H359L. Together, these results underscore the critical roles of ASFV *C315R* and *H359L* in virus transcription, DNA binding and control of DNA transcription into mRNA, which play an important role in ASFV replication.

### 3.5 Effects of ASFV-C315R and H359L expression on cell proliferation

The impact of C315R or H359L proteins on cell proliferation was assessed using CCK-8 assays after transfecting HA-C315R or HA-H359L plasmids into PK15, 3D4/21, MA104 and HEK293T cells for 24 or 48 h. The results demonstrated a significant decrease in cell viability in cells overexpressing the C315R or H359L proteins compared to the control groups (Supplementary Figure S3A), and with a dose-dependent manner (Supplementary Figure S3B), suggesting that C315R and H359L proteins may negatively impact cell viability.

### 3.6 Effect of C315R and H359L for PKR and eIF2α phosphorylation status and SG assembly

**Figure 6:**
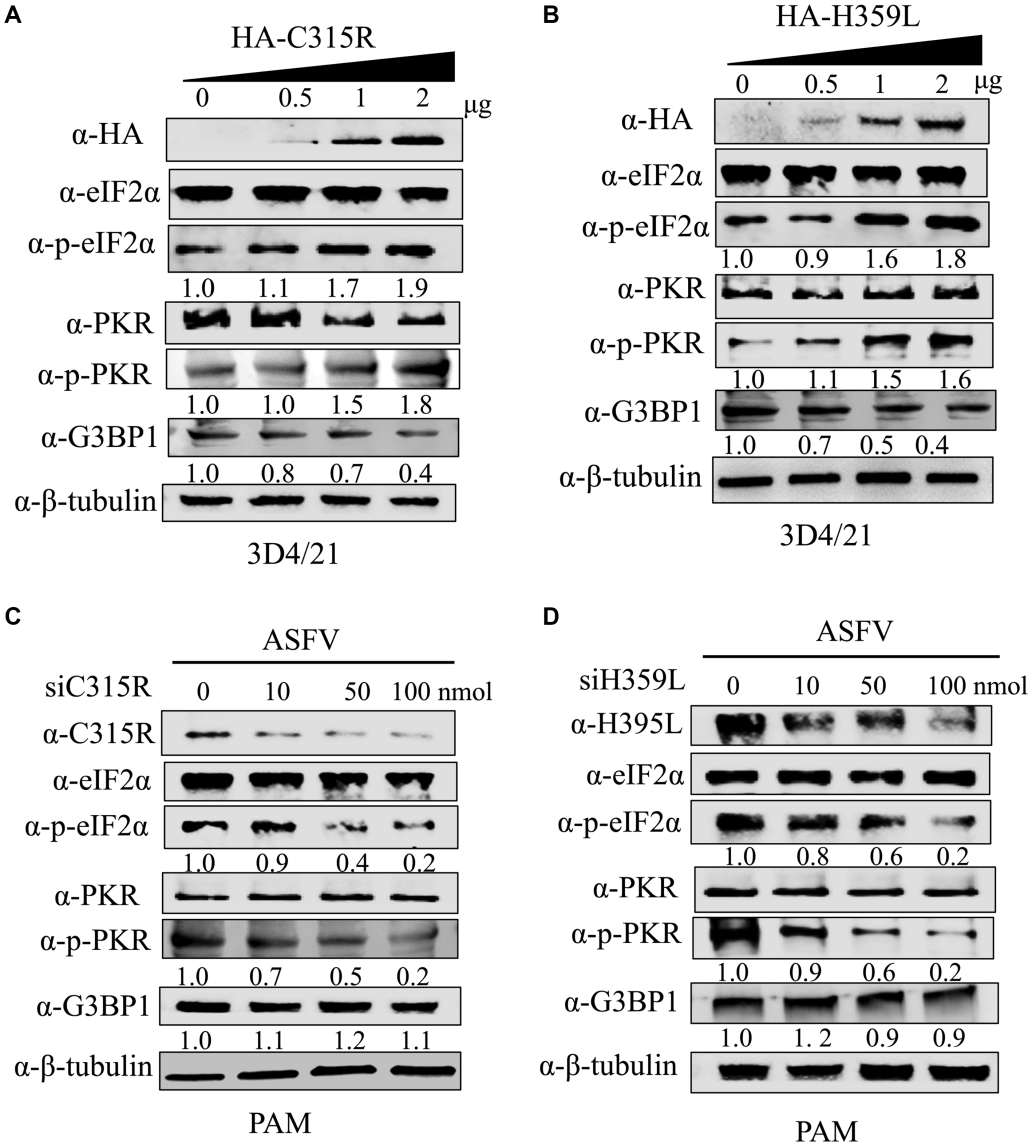
Effect of C315R and H359L for PKR and eIF2α phosphorylation status and SG assembly. 3D4/21 cells were transfected with HA-C315R or HA-H359L plasmids (0.5, 1, and 2 μg/mL, respectively) for 24 h, and followed the ASFV infection for 24 h. Empty vector (Vec) was included as negative control. Cells were harvested and processed for western blot analysis using 30 μg total protein per lane **(A,B)**. PMAs cells were transfected with siRNA targeting C315R or H359L plasmids (0, 10, 50, and 100 nM, respectively) for 24 h. Cells were harvested and processed for western blot analysis using 30 μg total protein per lane. C315R, H359L, p-PKR, PKR, p-eIF2α, eIF2α, and G3BP1 were detected with corresponding antibodies. *β*-Tubulin was probed as a control. The signals of p-PKR or p-eIF2α protein bands were determined by Image J. The intensities of p-PKR or p-eIF2α were normalized to total PKR or eIF2α **(C,D)**.

The host dsRNA-activated protein kinase R (PKR) is a pivotal component of the innate antiviral response. Activation of PKR during viral infection leads to the phosphorylation of a subunit of the eukaryotic translation initiation factor 2 (eIF2a), resulting in the inhibition of protein translation (Sadler et al., 2009). In our study, we explored the effects of overexpression HA-C315R and HA-H359L on host protein synthesis mechanisms. As shown in results, our findings indicate that the levels of phospho-PKR gradually increased in a dose-dependent manner alongside increased expression of HA-C315R and HA-H359L. Accordingly, an increase of phospho-eIF2α was observed in cells overexpressing HA-C315R or HA-H359L, suggesting that C315R or H359L activation triggers the PKR/eIF2α pathway (Figures 6A,B). Interestingly, we observed a slight decrease in the expression level of G3BP1, a marker of stress granule, although the underlying mechanism remains unclear. Conversely, higher concentrations of siRNA targeting ASFV C315R or H359L genes led to a notable decrease in both p-PKR and p-eIF2α, indicating that silencing these genes reduces the activation of the PKR/eIF2α pathway. In parallel, the expression of G3BP1 did not show significant alterations (Figures 6C,D). These results collectively suggest that ASFV C315R and H359L may induce host translation shutoff, relying on the activation of the PKR/eIF2α pathway, and that ASFV-C315R or-H359L is crucial for preventing eIF2α-independent SGs formation.

### 3.7 The viral protein C315R and H359L inhibits cytokine production in 3D4/21 cells

The inflammatory response plays a crucial role in immune responses against pathogens invasion (Tisoncik et al., 2012). This process involves a delicate balance between pro-inflammatory and anti-inflammatory cytokines, where an excessive immune response can be detrimental (Wang et al., 2020). In the context of viral infections, different viruses can either induce or suppress the expression of inflammatory factors.

**Figure 7:**
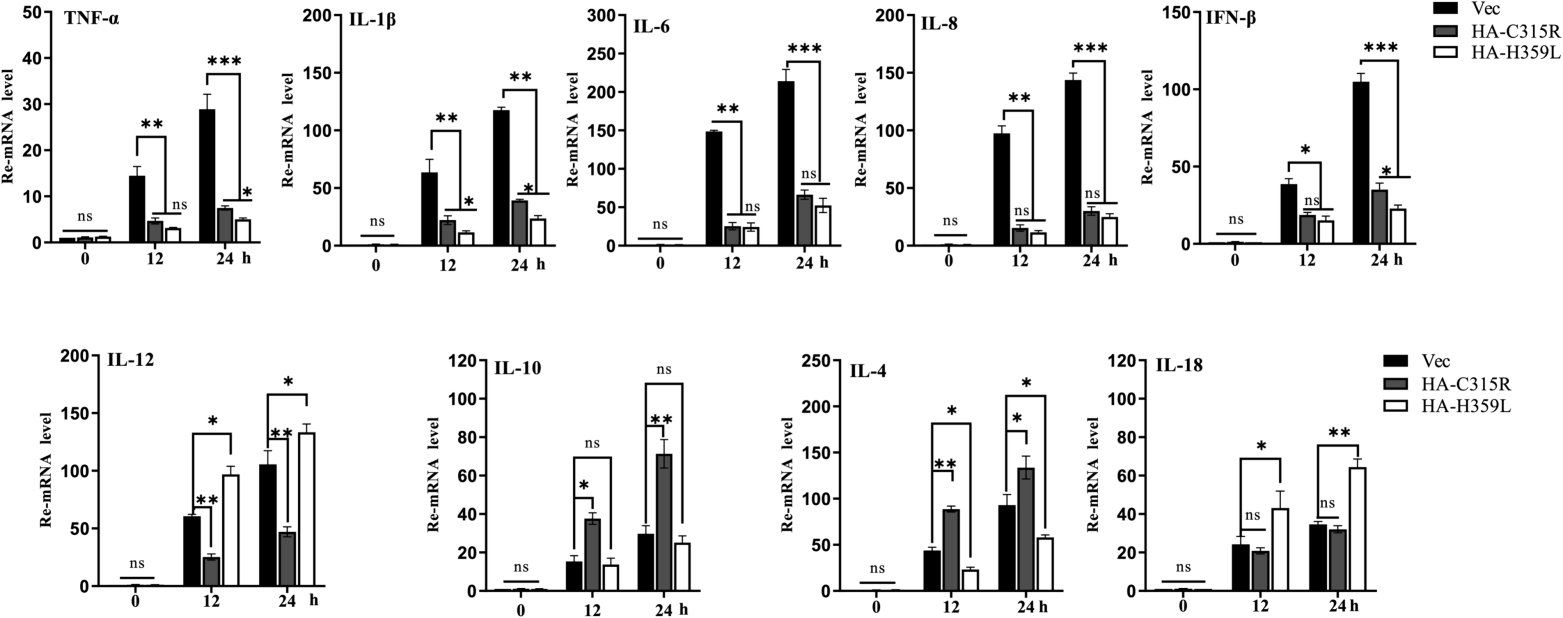
Overexpression of C315R or H359L induces the lower inflammatory cytokines production in 3D4/21. 3D4/21cells were either with or without transfected with ASFV-C315R or H359L at equal amounts of plasmids for 12 hpt, followed ASFV infection for 12 hpi. The mRNA expression levels of TNF-a, IL-1b, IL-8, IFN-β, IL-12, IL-10, IL-4, and IL-18 in the cells were detected by quantitative RT-PCR. Statistical significance is denoted by asterisks (*p < 0.05, **p < 0.01 determined by two-tailed Student’s *t*-test. ns, no significance).

To evaluate the impact of ASFV-C315R and-H359L on the inflammatory response, 3D4/21cells were transfected with equal amounts of ASFV-C315R or-H359L plasmids or left un-transfected. At 24 h post-transfection (hpt), the mRNA expression levels of various cytokines including TNF-a, IL-1 *β*, IL-8, IFN-β, IL-12, IL-10, IL-4, and IL-18 were measured using quantitative RT-PCR. The results indicated that neither ASFV-C315R nor ASFV-H359L stimulated the significantly transcription of TNF-a, IL-1β, IL-6, IL-8, and IFN-β compared to the vector control. Conversely, the expression of IL-12 and IL-18 was higher induced by ASFV-H359L, while IL-10 and IL-4 mRNA levels were increased under the same conditions (Figure 7). Notably, IL-18, known to play a protective role in many viral infections (Keyel, 2014), was specifically elevated by ASFV-H359L but not by ASFV-C315R. Additionally, the expression of the anti-inflammatory cytokines IL-10 and IL-4 was upregulated in ASFV-H359L-expressing cells but decreased in ASFV-C315R-expressing cells (Figure 7).

In summary, the expression of most pro-inflammatory cytokines was reduced under the influence of both ASFV-C315R and ASFV-H359L, suggesting a potential modulatory effect of these viral proteins on the host’s inflammatory response. This finding highlights the complex interactions between ASFV proteins and host immune mechanisms, which could have implications for the development of ASFV infection strategies and therapeutic approaches.

## 4 Discussion

Transcription is an important step in the expression of gene information in living organisms, significantly impacting virus-related diseases. TFIIB is a universal transcription factor, is integral to the transcription mechanism of RNA polymerase II (RNAPII) and aids in transcriptional termination (O’Brien and Ansari, 2022). In eukaryotes, RNAPII is recruited into DNA through a series of universal transcription factors (GTFs). And then RNAPII along with the GTFs form the preinitiation complex (PIC). These GTFs sequentially recruits TFIID, TFIIA, TFIIB, TFIIF, TFIIE, and TFIIH to the promoter. Among them, TFIIB is a single subunit GTF and a critical component of the PIC. It facilitates transcription by interacting with both DNA elements and other proteins. Moreover, it acts as a bridge connecting TFIID, which is bound to the DNA and RNAPII. The carboxy-terminal core domain of TFIIB interacts with the promoter DNA, TATA binding protein (TBP) and TFIIF, while its amino-terminal zinc ribbon domain interacts with the catalytic center of RNAPII. The interaction between the amino and carboxy-terminal domains of TFIIB enables it to form either an open or closed conformation, thereby stimulating transcription initiation (O’Brien and Ansari, 2022).

The ASFV has evolved a transcriptional regulatory protein, pC315R, which exhibits a three-dimensional structure highly similar to the host’s TFIIB, particularly in crucial conserved regions such as the B-band, B-cell cycle protein, and B-reading domain. These similarities suggest that pC315R might engage TFIIB-like functions to facilitate viral transcription. ASFV pC315R not only shows moderate similarity with the homologs of the transcription factor TFIIB from mimiviruses and large unclassified DNA viruses (Iyer et al., 2006) but also utilizes properties akin to TFIIB to express its genes while selectively inhibiting host antiviral genes, highlighting TFIIB as a potential target for antiviral therapy.

In eukaryotes, RPB3 serves as the core subunit of RNA polymerase II (Pol II), forming a heterodimer with the RPB11 subunit and is considered as a functional counterpart of the bacterial alpha subunit homodimer involved in promoter recognition. Notably, RPB1, RPB2, RPB3 and RPB11 are critical for the catalytic activity of Pol II RNA (Lee and Young, 2000). In ASFV, proteins responsible for replication, repair, and transcription are predominantly synthesized by the virus itself. The ASFV RNA polymerase shows a greater resemblance to eukaryotic Pol II than to those found in members of the NCLDV, suggesting sophisticated evolutionary adaptations. The pH359L enzyme, implicated in mRNA transcription, shares a high degree of similarity with the eukaryotic of the Pol II subunit 3(RPB3) (Iyer et al., 2006; Zhang et al., 2012). Together, we speculate that the pC315R and pH359L may play an important role in ASFV replication and transcription.

In this study, we investigate the roles of ASFV pC315R and pH359L in the viral infection cycle, focusing on expression characterization, host cell viability, translation machinery and inflammatory response. Our findings reveal that pC315R and pH359L induce host translation shutoff, dependent on the activation of the PKR/eIF2a pathway, and suppress inflammatory response by inhibiting cytokine production, thereby resisting the host’s antiviral defenses. We firstly showed that ASFV C315R and H359L existed in all ASFVs and was highly conserved across ASFV genotypes, and the phylogenetic relationship in different genotypes was consistent with the distribution of ASFV genotyping. Analysis of secondary structure and tertiary structures reveals no transmembrane region or signal peptide in pC315R and pH359L (data not shown), with their tertiary structure resembling those of TFIIB and RPB3, which was consistent with previous study (Rodríguez and Salas, 2013). Notable, the expression of C315R and H359L genes in spleen was highest compared with those in other different tissues from infected pigs, suggesting their potential as specific markers for ASFV infection detection.

The ASFV gene expression is categorized into several stages: immediate early, early, intermediate, and late genes. Immediate early and early genes are expressed before DNA replication begins, while intermediate and late genes are expressed post-DNA replication (Rodríguez and Salas, 2013; Cackett et al., 2020a; Cackett et al., 2020b). In this study, we observed that the mRNA of *C315R* is expressed at 4 hpi, similar with *CP204L* (P30), whereas *H359L* expression begins at 12hpi, suggesting that *C315R* functions predominantly during the early phase, while H359L operates during the intermediate phase of the infection cycle, when viral DNA is extensively replicated and transcribed. Furthermore, under the influence of the DNA replication inhibitor AraC (Zhao et al., 2007), the expression levels of pC315R, pH359L, and P30 were consistent, unaffected by Arac. The expression of the control p72 protein was almost completely suppressed, suggesting that ASFV-pC315R and-pH359L are synthesize post-viral DNA replication (Figure 3). Our findings suggest that ASFV *C315R* is an early gene while *H359L* is the intermediate protein in high virulent ASFV genotype II CN/SC/2019, slightly later than P30 expression. These characteristics of *C315R* was consistent with previous study, which indicated that *C315R* is classified as an early gene in the high virulent ASFV strain genotype II Georgia 2007/1 (GRG), but not in the attenuated strain BA71V (Cackett et al., 2022).

The ASFV is a complex double-stranded DNA virus that primarily replicates in the cytoplasm of swine macrophages (Xian and Xiao, 2020). Determining the subcellular localization of pC315R and pH359L provides insights into their functional roles. To explore the characterization of pC315R and pH359L during ASFV infection, the expression and subcellular localization of pC315R and pH359L were evaluated upon ASFV infection in PAMs by Western blotting, using *β*-tubulin were considered nuclear and cytosolic protein control. We found that the expression of pC315R and pH359L mainly in the cytoplasm at 24 hpi (Figure 3D). These results underline the cytoplasmic replication nature of ASFV and suggest specific roles of these proteins in the viral lifecycle.

A previous study has suggested that there is a brief nuclear phase for ASFV, most of the replication and all viral morphogenesis occur in the cytoplasm of the infected cell (Ballester et al., 2011). Further research has shown that the ASFV C315R protein is associated with genes involved in protein transport between the endoplasmic reticulum (ER) and the Golgi apparatus, such as NOP58 ribonucleoprotein (NOP58), basic leucine zipper nuclear factor 1 (BLZF1), sorting linker protein 16 (SNX16), SDA1 domain containing 1 (SDAD1), and nuclear autoantigen sperm protein (NASP) (Lv et al., 2022). It suggests that the C315R interacted with other proteins encoded by host and played a crucial role in the host cell’s protein transport system, potentially influencing virus infection and the host’s antiviral response. However, the specific mechanisms by which C315R contributes to the protein transport signaling pathway require further investigation.

The viral titter, gene transcription, and protein expression of ASFV were significantly increased following transfection with HA-C315R or HA-H359L. Conversely, HAD_50_, qPCR and Western blot analysis revealed lower transcription levels of the ASFV *CP204L* early gene, *B646L* late gene in experiments using siRNAs targeting ASFV-C315R or ASFV-H359L. These findings showed the critical roles of C315R and H359L in ASFV transcription, functioning as non-redundant regulators on viral gene mRNA synthesis. These data suggested that *C315R* and *H359L*, acting as transcription factors and subunit analogs of EUkaryotic RNA polymerase, are integral to the regulation of viral replication and transcription (Nikolov and Burley, 1997).

Several lines of evidence indicate that ASFV genes (MGF110-7 L, pE66L) modulate the PKR/eIF2a pathway and the formation of stress granules to counteract the host’s antiviral response (Zhong et al., 2022; Shen et al., 2021). While ASFV infection generally induces low levels of inflammatory cytokines, the deletion of the ASFV-H240R gene has been shown to reduce virus growth and trigger high level of inflammatory cytokines (Zhou et al., 2022). Our data demonstrate that both *C315R* and *H359L* decrease cell viability and induce host translation shutoff, relying on the activation of the PKR/eIF2α pathway, suggested a regulation of the antiviral response at the translation stage. ASFV C315R or H359L prevents the eIF2α-independent SGs formation. SG formation is part of the integrated stress response (ISR), which have been shown to regulate and control cytokine mRNA aggregation and expression (Svenja et al., 2019). Additionally, overexpression of ASFV C315R or H359L significantly reduces the most of cytokine’s expression. Notably, we observed that pC315R or pH359L reduced the inflammatory response, thereby inhibited the host’s defense against ASFV infection (Figure 7). We speculate that the observed growth defect in ASFV infection may be due to the suppression of the inflammatory signaling pathway by ASFV C315R or H359L, facilitating virus replication. Specifically, the reduced cytokines production in cells overexpressing C315R or H359L, including IL-1*β*, TNF-a, IL-6, IL8, and IFN-β, appears to promote ASFV replication.

Targeted gene deletion from the ASFV genome has been a powerful tool to study gene function, particularly regarding virus replication and virulence. The attenuated viruses have been shown effective in preventing disease during challenges with parental virulent strains (Chen et al., 2020; O’Donnell et al., 2015b; O’Donnell et al., 2015a; Borca et al., 2020; O’Donnell et al., 2017). In our study, a recombinant ASFV lacking the pC315R or pH359L gene (ASFV-∆C315R or ASFV-∆H359L) was trying to construct but failed (data not shown). Whether the C315R and H359L gene are indispensable for virus replication and thus the virus could not survive after losing these two genes need to be further studied in future.

**Figure 8:**
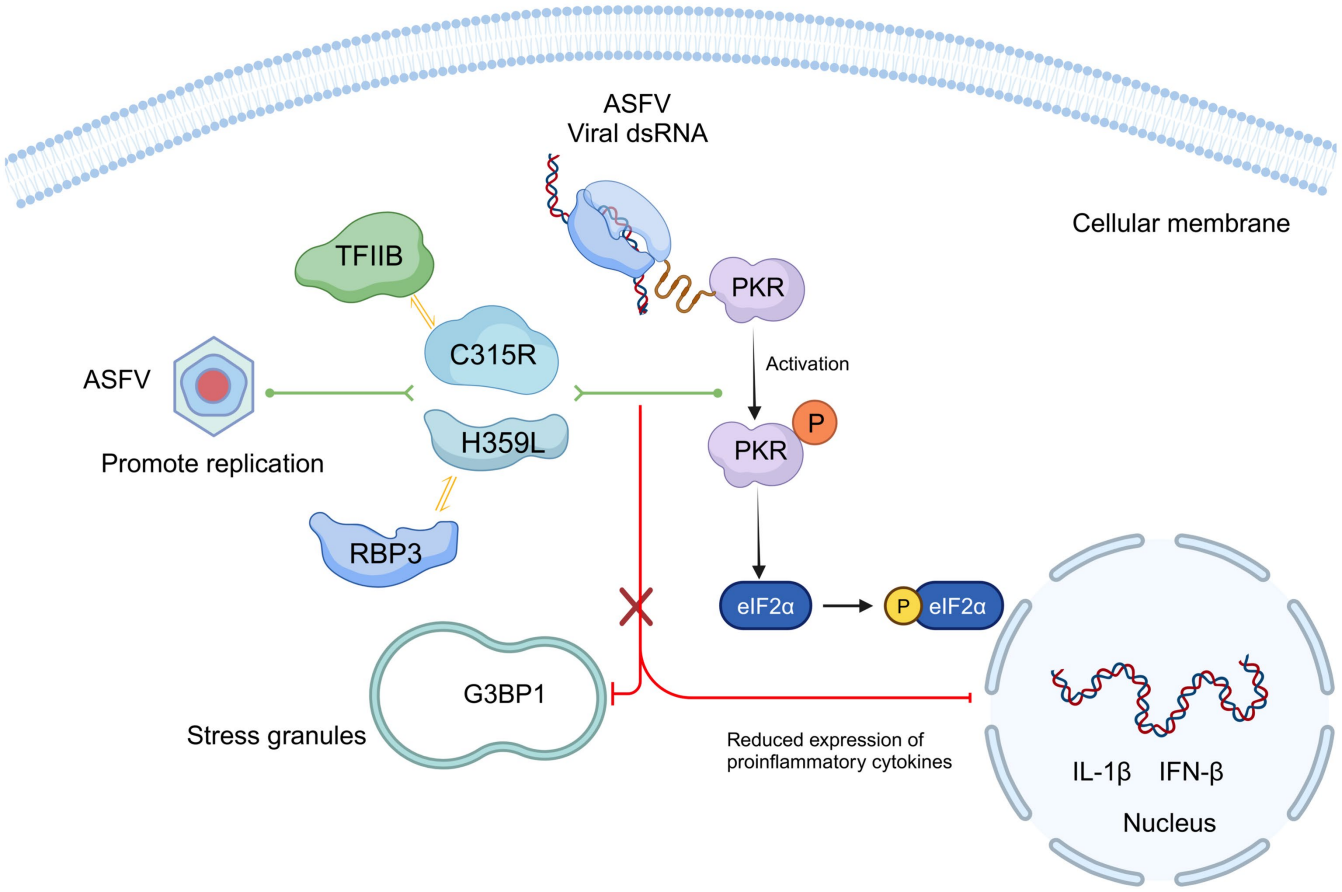
Schematic model of ASFV C315R and H359L inhibition host translation by activating the PERK/PKR-eIF2a pathway and suppression inflammatory response. Mechanistically, C315R and H359L decrease cell viability and induce host translation shutoff, relying on the activation of the PKR/eIF2α pathway. Additionally, ASFV C315R or H359L prevents the eIF2α-independent SGs formation and overexpression of ASFV C315R or H359L significantly reduces the most of cytokine’s expression.

Our findings highlight the primary functions and characteristics of ASFV pC315R and pH359L during viral infection. We supposed that C315R and H359L decrease cell viability and may induce host translation shutoff, relying on the activation of the PKR/eIF2α pathway. Additionally, ASFV C315R or H359L prevents the eIF2α-independent SGs formation and overexpression of ASFV C315R or H359L significantly reduces the most of cytokine’s expression (Figure 8). Future studies are essential to further delineate the functional relationship between ASFV pC315R or pH359L and the host.

## Data Availability

All data generated or analyzed in this study are included in this article and its Supplementary material.

## Supplementary material

SUPPLEMENTARY FIGURE S1 Subcellular localization of C315R **(A)** and H359L **(B)**. MA104 cells were transfected with C315R and H359L plasmids, then, the cells were collected after transfection 12 h or 24 h, the expression and localization of C315R and H359L protein were evaluated by confocal microscopy using anti-HA tag antibodies.

SUPPLEMENTARY FIGURE S2 Identification of expression of pCDNA3.1-HA-C315R or pCDNA3.1-HA-H359L plasmids in HEK293T, MA104 and PK15 cell lines.

SUPPLEMENTARY FIGURE S3 Effect of C315R and H359L on cell proliferation. HEK293T, PK15, MA104, or 3D4/21 cells were transfected with empty vector (Vec) or HA-C315R or H359L plasmid for 24 or 48 h **(A)**. 3D4/21 cells were transfected with empty vector or HA-C315R or HA-H359L plasmids with 0, 0.25, 0.5, 1, and 2 μg/mL, respectively **(B)**. Cell viability was assessed with CCK-8 assays. Data were shown as mean ± SD based on three independent experiments. Statistical significance is denoted by asterisks (**p* < 0.05, ***p* < 0.01, ****p* < 0.001 determined by two-tailed Student’s *t*-test. ns, no significance).
