# Supplementary figures and images for: African swine fever virus RNA polymerase subunits C315R and H359L inhibition host translation by activating the PKR-eIF2a pathway and suppression inflammatory responses
Source: Front Microbiol. 2024 Sep 24;15:1469166. doi: 10.3389/fmicb.2024.1469166 (PMC11458487; doi:10.3389/fmicb.2024.1469166)

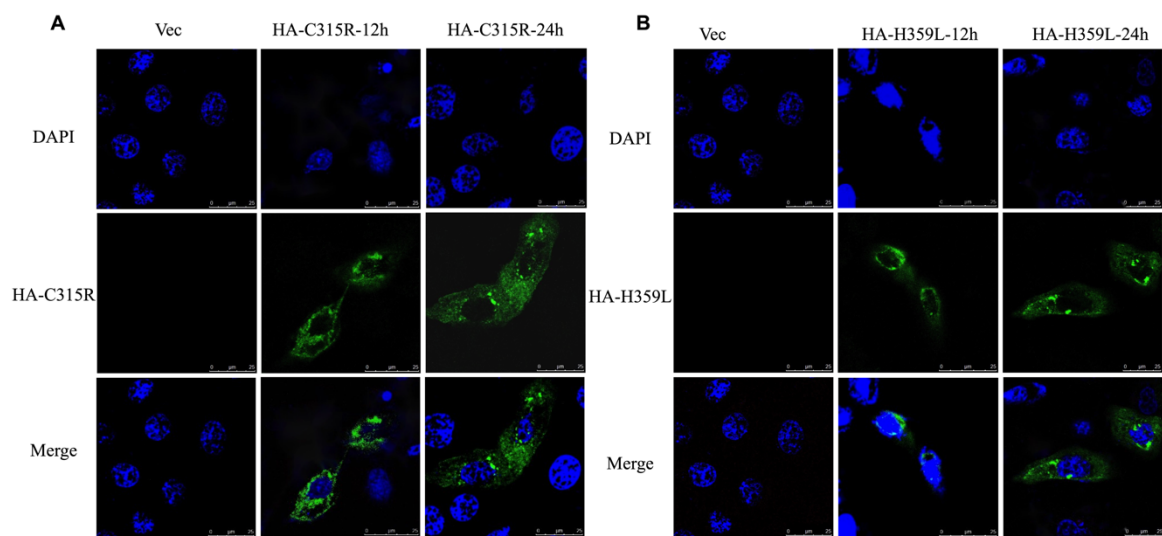

Figure S1

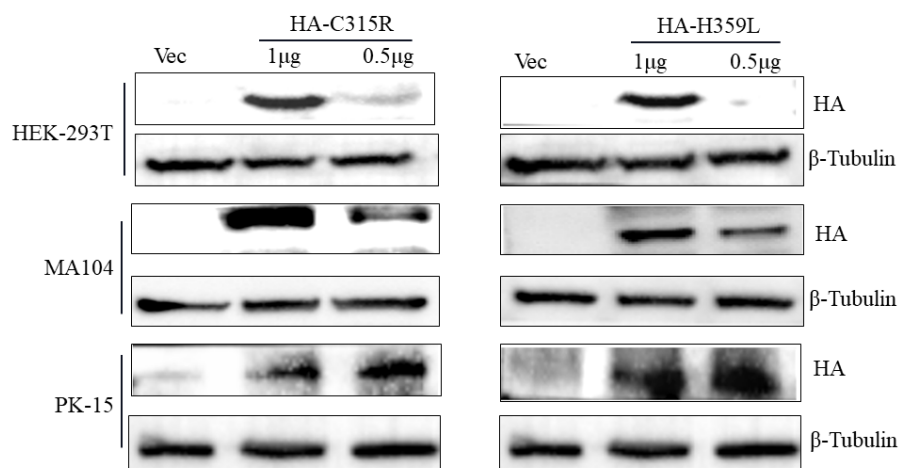

Figure S2

**A**

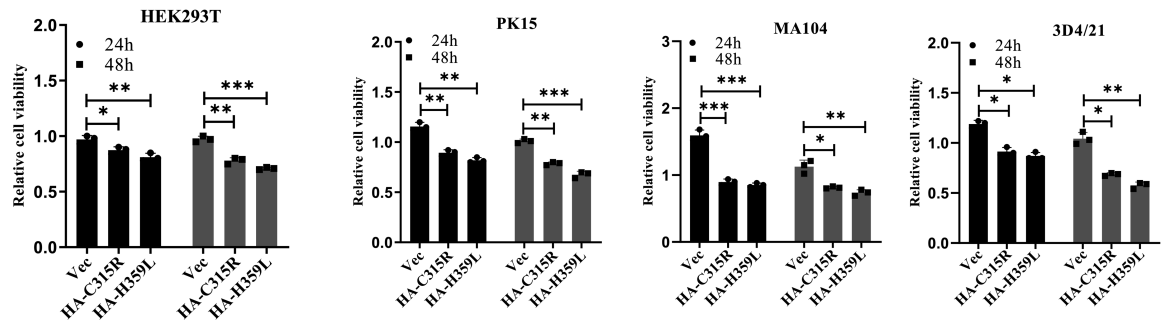

**B**

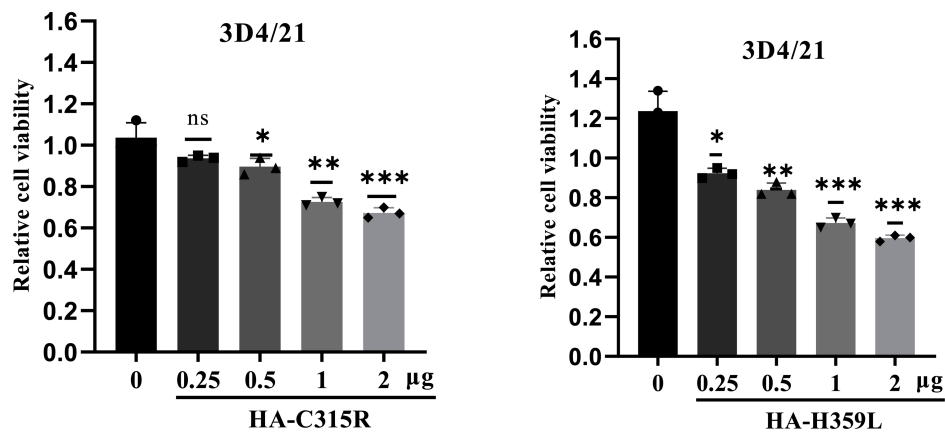

Figure S3

Supplement: Supplementary file 1 [file Data_Sheet_1.PDF]
