# Supplementary material for: African swine fever virus RNA polymerase subunits C315R and H359L inhibition host translation by activating the PKR-eIF2a pathway and suppression inflammatory responses
Source: Front Microbiol. 2024 Sep 24;15:1469166. doi: 10.3389/fmicb.2024.1469166 (PMC11458487; doi:10.3389/fmicb.2024.1469166)
Supplement: Supplementary file 2 [file Data_Sheet_2.pdf]

**Table S1. Gene homology comparison of C315R or H359L in different ASFV strains**

| No. | Name | Accession numbers | Genotypes | Percent identity of nucleotide (%) | | Percent identity of amino acids (%) | |
|---|---|---|---|---|---|---|---|
| | | | | C315R | H359L | C315R | H359L |
| 1 | SY18 | MH766894.1 | II | 100 | 100 | 100 | 100 |
| 2 | AnhuiXCGQ | MK128995.1 | II | 99.9 | 100 | 100 | 100 |
| 3 | HLJ/2018 | MK333180.1 | II | 99.9 | 100 | 100 | 100 |
| 4 | CAS19-01 | MN172368.1 | II | 99.9 | 100 | 100 | 100 |
| 5 | wbBS01 | MK645909.1 | II | 99.9 | 100 | 100 | 100 |
| 6 | Georgia 1 | MH910495.1 | II | 99.9 | 100 | 100 | 100 |
| 7 | Pol16 | MG939583.1 | II | 99.9 | 100 | 100 | 100 |
| 8 | Belgium/wb | MK543947.1 | II | 99.9 | 100 | 100 | 100 |
| 9 | Belgium18/1 | LR536725.1 | II | 99.9 | 100 | 100 | 100 |
| 10 | Georgia 2 | MH910496.1 | II | 99.9 | 100 | 100 | 100 |
| 11 | Georgia 3 | FR682468.1 | II | 99.9 | 100 | 100 | 100 |
| 12 | LT14/1490 | MK628478.1 | II | 99.9 | 100 | 100 | 100 |
| 13 | Estonia 2014 | LS478113.1 | II | 99.9 | 100 | 100 | 100 |
| 14 | Czech | LR722600.1 | II | 99.9 | 100 | 100 | 100 |
| 15 | Moldova | LR722599.1 | II | 99.9 | 100 | 100 | 100 |
| 16 | Kyiv/2016 | MN194591.1 | II | 99.9 | 100 | 100 | 100 |
| 17 | LT14/1490 | MK628478.1 | II | 99.9 | 100 | 100 | 100 |
| 18 | HuB20 | MW521382.1 | II | 99.9 | 100 | 100 | 100 |
| 19 | Benin 97/1 | AM712239.1 | I | 99 | 97.6 | 100 | 98.9 |
| 20 | 26544/OG10 | KM102979.1 | I | 99 | 97.6 | 100 | 98.9 |
| 21 | 47/Ss/2008 | KX354450.1 | I | 98.9 | 97.6 | 100 | 100 |
| 22 | LIV_5_40 | MN318203.1 | I | 99 | 92.3 | 100 | 94.4 |
| 23 | OURT 88/3 | AM712240.1 | I | 99 | 97.7 | 100 | 98.9 |
| 24 | E75 | FN557520.1 | I | 98.9 | 97.7 | 100 | 98.9 |
| 25 | BA71 | KP055815.1 | I | 99 | 97.7 | 100 | 98.9 |
| 26 | R25 | MH025918.1 | IX | 95.1 | 94.2 | 98.7 | 95 |
| 27 | R8 | MH025916.1 | IX | 95.1 | 94.2 | 98.7 | 95 |
| 28 | N10 | MH025919.1 | IX | 95.1 | 94.1 | 98.7 | 100 |
| 29 | R35 | MH025920.1 | IX | 95.1 | 94.2 | 98.7 | 100 |
| 30 | Ken06.Bus | KM111295.1 | IX | 89 | 94.2 | 98.7 | 95 |
| 31 | Ken05/Tk1 | KM111294.1 | X | 89.4 | 94.9 | 98.7 | 100 |
| 32 | LIV_5_40 | MN318203.1 | I | 99 | 92.3 | 100 | 94.4 |
| 33 | RSA_2 | MN336500.1 | XXII | 93.2 | 93.6 | 83.8 | 86.4 |
| 34 | Warmbaths | AY261365.1 | III | 94.5 | 95.7 | 94.2 | 92.8 |
| 35 | Warthog | AY261366.1 | IV | 94.7 | 95.2 | 94.5 | 93.6 |
| 36 | Tengani 62 | AY261364.1 | V | 93.5 | 94.6 | 92.3 | 92.8 |

**Table S2. List of primers or probes used in this study**

| Purpose | Gene | Sequence (5′-3) |
|---|---|---|
| qPCR | *H359L* | F: AGGATTCCACGGACCTGTTT<br>R: TTTAAGCTTAGGGCCTGCCA<br>FAM-CCGCAGAGCAAATACCAGTGTCTCGT-TAMRA |
| | *C315R* | F: GGATCTTCTGCGCTCCCTAT<br>R: CGCCGATGTTCTTCTCATCC<br>FAM-ACAAATCCACCAAGAACTGCAGGAGGA-TAMRA |
| | *CP204L* | F: GCGGTAGAATTGTTACGACCGCT<br>R: CCTCCGATGAGGGCTCTTGC<br>FAM-ACGGAATCCTCAGCATCTTCGGA-TAMRA |
| | *B646L* | F: CTGCTCATGGTATCAATCTTATCGA<br>R: GATACCACAAGATCRGCCGT<br>FAM-CCACGGGAGGAATACCAACCCAGTG-TAMRA |
| | *GAPDH* | F: TGGAAAGGCCATCACCATCT<br>R: ATGGTCGTGAAGACACCAGT<br>FAM-CCAGGAGCGAGATCCCGCCA-TAMRA |
| | *TNFα* | F: GGC TGC CTT GGT TCA GAT GT<br>R: CAG GTG GGA GCA ACC TAC AGT T |
| | *IL-1β* | F: AGGGACATGGAGAAGCGATTT<br>R: TTCTGCTTGAGAGGTGCTGATG |
| | *IL-6* | F: GGCCATTCGGATAATGTAGCT<br>R: GTGTCCTAACGCTCATACTTT |
| | *IL-8* | F: TTCCTGCTTTCTGCAGCTCTCT<br>R: GGGTGGAAAGGGTGTGGAATG |
| | *IFN-β* | F: AGTTGCCTGGGACTCCTCAA<br>R: CCTCAGGGACCTCAAAGTTCAT |
| | *IL-12* | F: ATTGAGGTCGTGCTGGAAGCT<br>R: AGATTCTTGGGAGGGTCTGGTT |
| | *IL-10* | F: GCCTTGTCAGAGATGATCCAGTT<br>R: TTCTCCCCCAGGGAGTTCAC |
| | *IL-4* | F: GTACCAGCAACTTCGTCCAC<br>R: AAGGTTTCCTTCTCCGTCGT |
| | *IL-18* | F: GCAGTAACCATCTCTGTGCAGTGTA<br>R: TCATCAATATTATCAGGAGGACTCATTT |
| Recombinant plasmids construction | *C315R* | F:CGC<u>CATATG</u>GATGCCCTATTAAAGGAAATAGAAAAGTTATCG<br>R:CCG<u>GAATTC</u>TTAAATTAATTTTTTTGCCGCGTTTAAATGATACTGC |
| | *H359L* | F:CGC<u>CATATG</u>GAAAAAATTTTCCAAAACGTGGAAATCAAAC<br>R:CCG<u>GAATTC</u>TTAAGCAATCAGTTCATCAACATTTTTTTCAAGAATTTG |

| | | |
|---|---|---|
| siRNA | *H359L-1* | Sense: CCGCUAAGCGCCAUAUAUUTT<br>Antisense: AAUAUAUGGCGCUUAGCGGTT |
| | *H359L-2* | Sense: GCUCUGCUAAUGCCCUUAATT<br>Antisense: UUAAGGGCAUUAGCAGAGCTT |
| | *H359L-3* | Sense: GUAGGAAGCCAAUCUCAAATT<br>Antisense: UUUGAGAUUGGCUUCCUACTT |
| | *C315R-1* | Sense: GGACUGGUUUGAACCUGAATT<br>Antisense: UUCAGGUUCAAACCAGUCCTT |
| | *C315R-2* | Sense: GGAGCUCAAAUCCCUAAAUTT<br>Antisense: AUUUAGGGAUUUGAGCUCCTT |
| | *C315R-3* | Sense: GUACCUACAGUGCCUUACATT<br>Antisense: UGUAAGGCACUGUAGGUACTT |
